# Effect of Natural Disasters on Youth Mental Health: A Systematic Review

**DOI:** 10.1007/s40653-026-00853-y

**Published:** 2026-03-26

**Authors:** Raquel María González García, Helal Uddin, Tatiana Cuartas-Álvarez, Esther González García, Nur A Habiba Mukta, Rafael Castro-Delgado

**Affiliations:** 1 Department of Medicine, University of Oviedo, Oviedo, Spain; 2 Department of Sociology, East West University, Dhaka, Bangladesh; 3 SAMU-Asturias-Health Service of the Principality of Asturias, Oviedo, Spain; 4 Research Group on Prehospital Care and Disasters (GIAPREDE), Health Research Institute of the Principality of Asturias, Oviedo-33001, Spain; 5 University of Castilla-La Mancha, Ciudad Real, Spain

**Keywords:** Natural disaster, Mental health, Anxiety, Depression, PTSD, Youth

## Abstract

Background Natural disasters have a significant impact on the mental health of affected populations, particularly children and young people. The reported risks have become more severe as environmental and climate challenges increase. Thus, it is imperative to identify vulnerable groups to design early warning and effective interventions. This study aimed to synthesize evidence on how natural disasters affect the mental health of young people (aged 15–24 years) and to explore factors influencing their vulnerability and resilience. Methods Four databases, namely PubMed, PsycINFO, SCOPUS, and Web of Science, were searched using pre-established keywords to find peer-reviewed English-language studies. Using predefined eligibility criteria, two reviewers independently examined the titles, abstracts, and full texts. Rayyan software was used for screening. For quantitative articles, we used the National Institutes of Health Quality Assessment Tool (NIH-QAT), and for qualitative studies, we used the Critical Appraisal Skills Program (CASP) checklist. Additionally, the Mixed Methods Appraisal Tool (MMAT) was applied to mixed-method studies. Results A total of 97 articles were extracted from the database search. 70 articles were assessed by title and abstract, and 58 were eligible for full-text review. Finally, 33 studies were included: 14 cohort studies, 16 cross-sectional studies, 1 qualitative study, and 2 mixed methods studies. Most were rated “good” in terms of methodological quality but had a significant risk of bias. The findings reported that young people exposed to natural disasters experience stress, anxiety, depression, PTSD, and suicidal ideation or behaviors. Identified risk factors included female gender, older adolescence, low socioeconomic status, direct disaster exposure, family member loss, displacement, lack of social support, and substance abuse. Resilience was variably defined and did not always indicate well-being, but in some contexts, it reflected forced or adaptive coping. Conclusions Natural disasters have an adverse impact on young mental health, increasing the risk of stress, anxiety, depression, PTSD, and suicidal thoughts. Strengthening community and school-based support systems, implementing psychological first aid, and ensuring access to early and sustained mental health interventions are critical. Long-term mental health outcomes are influenced by the availability of social, institutional, and healthcare resources. Reducing psychological harm requires coordinated efforts involving caregivers, educational institutions, health systems, and community stakeholders.

**Supplementary Information:**

The online version contains supplementary material available at https://doi.org/10.1007/s40653-026-00853-y.

## Abstract (structured)

**Key Practitioner Message:**

***What is known*:**

- Natural disasters have a significant impact on the mental health of affected populations, particularly children and young people.

***What is new*:**

- Using four databases without time and geographical restrictions, this systematic review synthesizes evidence from 33 studies to identify risk factors associated with youth mental health outcomes following natural disasters.
- The review also focuses on the role of resilience in youth recovery, an area that has been underexplored in previous systematic reviews.

***What is significant for clinical practice*:**

- Policies and interventions can be designed based on the identified by identifying risk factors (e.g., female gender, later adolescence, low socioeconomic status, direct exposure to the disaster, family member loss, displacement, lack of social support, and substance abuse), particularly among youth with compounded vulnerabilities.
- Psychosocial interventions should not only prioritize symptom reduction but also focus on long-term psychosocial support, caregiver engagement, and community-based recovery programs. Future research may focus on age-sensitive and culturally appropriate disaster-response strategies.

**Supplementary Information:**

The online version contains supplementary material available at https://doi.org/10.1007/s40653-026-00853-y.

##  Background

Natural disasters are events of geophysical, meteorological, hydrological, or biological origin that cause serious disruptions in the functioning of a community or society, exceed its response capacity, and generate significant human, material, economic, and environmental losses (Castro-Delgado & González, 2020; UNDRR, 2022). In recent decades, the frequency and intensity of these phenomena have increased significantly due to factors such as climate change, unplanned urbanization, environmental degradation, and population growth (IPCC, 2023). This increase not only intensifies physical and infrastructural damage but also generates severe consequences for public health – including increases in communicable diseases, injuries, forced displacement, and a deterioration in the mental and emotional well-being of affected populations (UNDRR, 2022); Amro et al., 2022). The health, economic, political, and social repercussions of these events can be devastating, both in the short and long term (World Health Organization, 2021). In many cases, local health systems are overwhelmed or weakened, making it difficult to provide adequate and timely care and triggering public health crises that may extend far beyond the initial event. Thus, natural disasters are not only environmental threats but also triggers health emergencies with multisectoral and persistent consequences.

Childhood and adolescence are critical stages of human development, in which biological, emotional, and social factors interact to shape mental health and long-term well-being. In the context of natural disasters, this population is particularly exposed to multiple forms of vulnerability (Lai, B. S., & La Greca, A., 2020). For example, the interruption of schooling, which plays an essential role in children’s well-being (Cuartas Alvarez T. et al., 2024), along with the loss of routines and forced displacement, affects not only their access to education but also their sense of stability and security, which are fundamental elements for healthy emotional development (UNICEF, 2022). Furthermore, exposure to traumatic events such as the loss of loved ones, physical injuries, or the destruction of one’s home significantly increases the risk of developing post-traumatic stress disorder (PTSD), depression, and anxiety, with effects that can persist for years if not adequately addressed (Pfefferbaum et al., 2015; Uddin et al., 2024). Consequently, natural disasters not only compromise the physical integrity of children and adolescents but also threaten their overall development and future mental health, making them one of the most vulnerable populations in the face of these emergencies. Hence, there is an urgent need to better understand vulnerable populations, such as youth, in order to enable early detection and appropriate interventions before and after disasters.

The development of mental disorders in young people exposed to natural disasters, such as post-traumatic stress disorder (PTSD), depression, anxiety, and suicidal ideation, is determined by a complex interaction of risk and protective factors. The main risk factors include the intensity and duration of exposure to the traumatic event, the loss of family members or loved ones, the collapse of support networks, a personal or family history of mental disorders, and precarious socio-economic conditions (Furr et al., 2010; NHS, 2021). Furthermore, exposure to multiple traumatic events and a lack of access to adequate mental health services significantly increase the likelihood of developing severe symptoms (Trickey et al., 2012). However, protective factors include stable emotional support from caregivers, family cohesion, continued education, early access to psychosocial interventions, and the strengthening of coping and resilience skills (Masten & Narayan, 2012). The presence of these conditions can significantly mitigate the impact of trauma, promote more effective recovery, and reduce the risk of lasting psychological consequences. Therefore, addressing mental health in post-disaster contexts must consider both the identification of specific risks and actively promoting protective factors at the individual, family, and community levels (Gibbs et al., 2021).

Although the body of research on the mental health effects of natural disasters has progressively grown in recent decades, studies focusing specifically on children and adolescents remain limited, and in many cases, fragmented in terms of study designs and procedures. Most studies have been conducted in contexts of high exposure or high-profile disasters, leaving out numerous local or prolonged experiences in regions with fewer research resources (NHS, 2021; Pfefferbaum & North, 2020). This is compounded by the lack of culturally adapted assessment tools, the limited number of longitudinal studies, the underrepresentation of vulnerable populations such as displaced or minority children, and the ethical difficulty of including control groups in emergency situations (Burt et al., 2018). Consequently, a notable gap persists in the scientific literature regarding the differentiated effects of natural disasters on youth mental health, as well as the contextual factors that moderate this vulnerability. This limitation not only hampers evidence-based policymaking but also obscures the psychological experiences of millions of affected young people. Therefore, there is an urgent need for a systematic review that synthesizes the findings from existing scientific studies and incorporates approaches sensitive to age, cultural context, and social determinants, in order to identify effective and equitable interventions.

Hence, considering the gap in the existing literature, this systematic review aims to synthesize how natural disasters affect the mental health of young people and to identify the risk factors associated with these mental health conditions. The study’s findings aim to inform the development of evidence-based mental health policies and interventions for youth in disaster-affected communities. By highlighting both vulnerabilities and protective factors, this review seeks to support the design of early and targeted intervention strategies that are developmentally appropriate and contextually sensitive. Ultimately, the goal is to contribute to a more resilient and better-supported young population in the face of increasing climate-related and environmental crises.

##  Methods

### Study Design

Following the guidelines of the Preferred Reporting Items for Systematic Reviews and Meta-Analysis (PRISMA) (Moher, Liberati, Tetzlaff, Altman, & The PRISMA Group, 2009), the protocol for this systematic review was drafted and registered under the code “CRD42025648903” in the International Prospective Register of Systematic Reviews (PROSPERO). Due to the heterogeneity in the measurement of the variables of interest (Table 2), we adopted a narrative synthesis method to report the study findings.

### Information Sources

The literature search to support this review was conducted between February and March 2025, using the following four bibliographic databases: PubMed, PsycINFO, SCOPUS, and Web of Science, irrespective of publication date or geographic location.

### Search Strategy

A bibliographic search strategy was developed using MeSH terms and by combining keywords such as natural disasters, mental health, and youth. We used Boolean operators (AND, OR, and NOT) along with truncations, in accordance with the specific guidelines and unique filters of each database.

### Eligibility Criteria

We applied predefined inclusion criteria to determine eligibility, such as studies in which the primary sample comprised individuals aged 15 to 24 years. We excluded studies that focused on man-made or technological disasters, war, pandemic events, or terrorism. No time limit was placed on publication date, and the search was confined to English-language sources. These inclusion and exclusion criteria are presented in detail in Table 1.

**Table 1:** Inclusion and exclusion criteria of the systematic review

	Inclusion criteria	Exclusion criteria
Population	Youth population aged 15 to 24 years	Adults (over 24 years) or children under 15 years
Intervention/exposure	Natural disasters	Disasters caused by human actions, wars, conflicts, and pandemic event
Comparison	None	
Outcome	Studies investigating mental health outcomes	Studies that focus on physical illnesses only
Study design	Peer-reviewed quantitative, qualitative, and mixed methods studies, including RCTs, non-RCTs, cohort studies, cross-sectional studies, and surveys	Protocols, commentaries, conference proceedings, opinion pieces, reviews, or systematic reviews
Settings	No restriction on location and geographic setting	

### Screening Procedures

Following the initial systematic search, all retrieved records were imported into the Rayyan (Qatar Computing Research Institute [QCRI]) software. After removing duplicates, two reviewers (R.G.G. and H.U.) independently screened the titles and abstracts of the retrieved articles using the established inclusion and exclusion criteria to identify studies eligible for full-text review. To select the final list of studies for inclusion, the same two reviewers then independently examined the full texts of the remaining articles. Additionally, a third reviewer (R.C.D.) was consulted to resolve any discrepancies encountered during the screening and reviewing phases.

### Data Extraction and Management

Two reviewers (R.G.G. and H.U.) independently extracted specific information from the included studies using a standardized Excel data extraction template. The template captured general bibliographic information (article title, author(s), year of publication, and year of study), study characteristics (objectives, design, countries involved, and sample size), and main methodological characteristics. A second section of the template included the primary mental health outcomes, associated factors, and key findings relevant to the objective of this study. We also recorded whether mental health outcomes were self-reported by youth or reported by parents or caregivers, noting that in several studies, mental health outcomes were reported by parents or caregivers acting as proxies for young people. This approach is commonly used when participants are younger children and direct assessment is not feasible, or when ethical and practical constraints are described by the original investigators. Following the data extraction conducted by the two reviewers, a third reviewer (R.C.D.) verified the data and resolved any discrepancies that arose.

### Quality Appraisal/risk of Bias

To evaluate the quality of the studies included in this analysis, we employed the National Institutes of Health Quality Assessment Tool (NIH-QAT) for quantitative studies, specifically those with a cohort or cross-sectional design, and used the Critical Appraisal Skills Program (CASP) checklist forqualitative studies. Furthermore, for two articles that incorporated both quantitative and qualitative methodologies, we applied the Mixed Methods Appraisal Tool (MMAT). The overall quality of the studies was classified as poor, fair, or good using the GRADE (Grading of Recommendations Assessment, Development, and Evaluation) methodology. The studies were assessed independently by two reviewers (R.G.G. and H.U.). Any discrepancies that arose during the quality assessment and rating process were resolved through collective discussion; if necessary, a third reviewer (R.C.D.) was consulted to resolve any unresolved conflicts.

### Data Synthesis

Following data extraction, we systematically organized and synthesized the findings from the included studies using a descriptive-narrative synthesis approach. The extracted results were grouped into predefined themes or categories aligned with the study objectives, including types of mental health outcomes, health conditioning factors, and factors associated with mental health outcomes, which were further categorized into six sub-themes. This approach was considered the most appropriate given the heterogeneity in the variable measurement, study design, and reporting. For this reason, neither a meta-analysis nor pooled estimates were conducted. R.G.G. prepared the data synthesis, which was shared and cross-checked with H.U. and R.C.D.

### Ethical Considerations

This systematic review used existing published articles without any personally identifiable information from study respondents. Hence, ethical approval from an institution or research ethics committee was not required.

### Patient and Public Involvement

This review did not involve patients or the public in its research design, reporting, or dissemination plan.

## Results

### Selection of Article

**Fig. 1:**
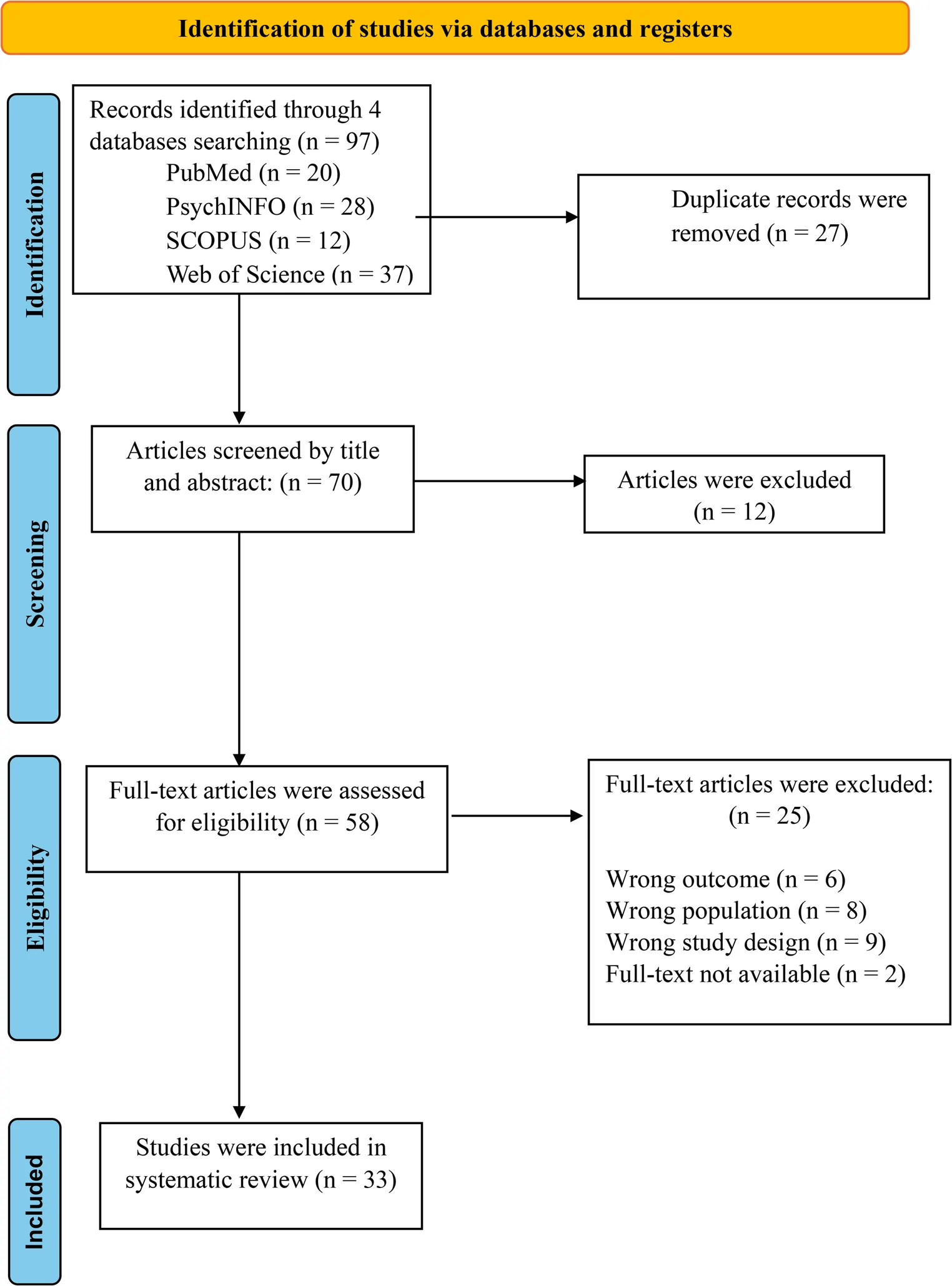
PRISMA flow diagram

A total of 70 unduplicated publications were identified. After reviewing the titles, 12 irrelevant publications (e.g., studies on man-made disasters, physical effects, and adult populations) were removed. The abstracts of the remaining 58 sources were examined, and 25 publications were eliminated due to ineligible study design (e.g., systematic reviews), inappropriate outcomes, ineligible populations, or unavailability of the full-text. The remaining 33 papers were read in full to identify studies meeting the inclusion criteria for the current analysis. The selection process is illustrated in the PRISMA flow diagram below (Fig. 1).

### Characteristics of the Studies Included in this Review

Characteristics of all included studies (*n* = 33) are presented in Table 2. More than half of the studies were published in the last six years (2019–2025), accounting for 57.5% of the total. Thirty studies (90.8%) were quantitative, of which 16 were cross-sectional, and 14 were cohort studies, while one was qualitative and two were mixed methods (quantitative and qualitative). The most frequently reported natural disaster was earthquakes (*n* = 12), followed by hurricanes and tornadoes (*n* = 10). Wildfires were cited in five cases, while floods and tsunamis appeared three times. Drought was mentioned only once (*n* = 1). Two studies did not specify the type of natural disaster examined.

**Table 2:** Characteristics of the studies included in this review

Study characteristics (*n* = 33)	Number of studies (%)
Year of publication (*n* = 33) 2006–2012 2013–2018 2019–2025	7 (21.2%) 7 (21.2%) 19 (57.5%)
Country (*n* = 33) United States Canada Australia Turkey China Nepal Indonesia Sri Lanka Japan Ecuador Puerto Rico Haiti Italy	9 (27.2%) 4 (12.1%) 4 (12.1%) 4 (12.1%) 3 (9%) 1 (3%) 1 (3%) 1 (3%) 1 (3%) 1 (3%) 1 (3%) 2 (6%) 1 (3%)
Study design (*n* = 33) Cross-sectional study Cohort study Qualitative study Mixed-method study	16 (48.4%) 14 (42.4%) 1 (3%) 2 (6%)
Types of natural disaster (*n* = 33) Earthquakes Hurricanes and tornadoes Wildfires Floods and tsunami Drought Not specified	12 (36.3%) 10 (30.3%) 5 (15.1%) 3 (9%) 1 (3%) 2 (6%)
Sample size (*n* = 33) < 100 100–500 500–2000 > 2000 Sample composition (*n* = 33) High school students University students Affected families (e.g., displacement, loss of housing or livelihood, injury or death of family members, or significant economic disruption) Others Sex (*n* = 23) Response rate (*n* = 5) Race/ethnicity (*n* = 5) African American Asian White/Caucasian Hispanic/Latino Other Not specified Type of mental health outcome (*n* = 33) Sleep problems Anxiety Depression PTSD Stress Suicidal ideation and behavior Hyperactivity disorder Coping styles and behavior Types of scales used for outcome measurement (*n* = 33) Depression Self-Rating Scale for Children (DSRSC) Patient Health Questionnaire- 9 (PHQ-9) Depression Anxiety Stress Scales-21 (DASS-21) Child PTSD Symptom Scale (CPSS) Child and Youth Resilience Measure (CYRM) Social Support for Adolescents Scale (SSAS) UCLA PTSD Reaction Index for Children and Adolescents Hospital Anxiety and Depression Scale (HADS) Strengths and Difficulties Questionnaire (SDQ) CRAFFT Questionnaire Author-developed measure Other scales*	4 (12.1%) 12 (36.3%) 11 (33.3%) 6 (18.2%) 18 (54.5%) 2 (6%) 12 (36.3%) 1 (3%) 55.14% Female **Mean**: 80.91%; **Range**: 61% − 99% 27.12% 1.6% 46.14% 18.36% 4.3% 2.4% 3 (9%) 14 (42%) 25 (75%) 16 (48%) 5 (15%) 8 (24%) 5 (15%) 3 (9%) 2 (3%) 5 (7%) 2 (3%) 4 (5%) 5 (7%) 3 (4%) 3 (4%) 3 (4%) 3 (4%) 3 (4%) 4 (5%) 27 (81%)

Sample sizes were generally large, with 36.3% of studies including between 100 and 500 participants, 33.3% including between 500 and 2,000 participants, and six studies (18.2%) including more than 2,000 participants. The United States (*n* = 9) and Canada (*n* = 4) accounted for most of the articles, followed by Australia (*n* = 4), Turkey (*n* = 4), and China (*n* = 3). Only five studies reported ethnicity or race, with Hispanic/Latino (46.14%) and African American (27.12%) as the predominant groups.

The most prevalent mental health outcomes were depression (75%), PTSD (48%), and anxiety (42%). It should be noted that several studies in this review identified multiple mental health outcomes, indicating the presence of comorbid conditions; hence, the reported diagnostic categories are not mutually exclusive. There was great variability in the types of scales used; the most frequently used were the Patient Health Questionnaire-9 (PHQ-9) depression scale (7%), the Child and Youth Resilience Measure (CYRM) (7%), and the Child PTSD Symptom Scale (5%).

### Types of Mental Health Conditioning Factors Following Natural Disasters

In this review, “mental health conditioning factors” refer to the conditions or experiences that can influence or modify youth mental health outcomes following natural disasters. These factors can be considered as secondary stressors that either intensify or mitigate the impact of disasters on mental health. Because these factors are particularly relevant, they are presented in a separate table **(**Table 3**)**, along with the number of studies that addressed each factor and the corresponding references.

**Table 3:** Types of mental health conditioning factors

Type of mental health conditioning factors (*n* = 33)	Number of articles reporting	References
Sex	12	(20) (21) (22) (23) (24) (25) (26) (27) (28) (29) (30) (38)
Age	8	(21) (26) (27) (30) (31) (37) (48) (51)
Race/ethnicity	5	(26) (33) (45) (50) (52)
Socioeconomic factors	7	(21) (22) (29) (30) (32) (33) (34)
Psychosocial support	13	(20) (21) (22) (25) (26) (28) (32) (34) (38) (44) (46) (49) (50) (51)
Disaster exposure	21	(20) (21) (22) (28) (30) (31) (32) (33) (34) (35) (36) (37) (38) (39) (40) (41) (42) (43) (45) (46) (52)
Multiple exposures	6	(25) (36) (42) (47) (48) (49)
Previous trauma	7	(26) (41) (42) (47) (48) (49) (50)
Self-esteem	5	(22) (23) (27) (28) (41)
Environmental and climate factors	3	(42) (43) (51)
Violence	4	(24) (25) (26) (52)
Substance use	4	(20) (41) (43) (51)

The most frequently reported mental health conditioning factors across the 33 included studies included sex, age, race, socioeconomic factors, psychosocial support, disaster exposure, multiple exposures, previous trauma, self-esteem, environmental and climate change, violence, and drug use/abuse. Additionally, the outcome measurement scales used in all included studies are listed in **Supplementary Table 1.**

### Main Psychological Impacts

The factors associated with anxiety, depression, PTSD, stress, and suicidal thoughts were extracted from all 33 studies and are presented in Table 4. These factors are divided into the following categories: socio-demographic and personal factors; exposure-related factors; psychological and behavioral factors; family and social support; media and information exposure; and structural and environmental risk factors, based on their themes and content.

**Table 4:** Factors associated with the main psychological impacts (*n* = 33)

Factors	Anxiety	Depression	PTSD		Stress	Suicidal ideation and behaviors	References
*Socio-demographic & Personal Factors*							
Female gender	*****	*******	*****		**	**	(20) (21) (22) (23) (24) (25) (26) (27) (28) (29) (30)
Older age	***	***	***		*		(21) (26) (27) (30) (31)
Race/ethnicity			*				(33)
Low socioeconomic status	*	**	****		***	*	(21) (22) (29) (30) (32) (33) (34)
***Exposure-Related Factors***							
Exposure to earthquake	*******	**********	*******		**	***	(20) (22) (24) (25) (28) (29) (30) (34) (35) (36) (37) (38)
Exposure to tornado		***	***			*	(21) (50) (52)
Exposure to hurricane	***	****	***		**		(26) (31) (32) (33) (39) (40)
Exposure to wildfire	***	***	**		**	**	(27) (41) (42) (43) (44)
Exposure to tsunami		*	*				(46)
Exposure to flood	*	**	*			*	(23) (42) (45)
Exposure to drought					*	*	(51)
Multiple exposures		***	***		*	*	(32) (36) (42) (47) (48) (49)
Family/relative or property loss	****	****	*****		*****	*	(21) (22) (28) (30) (31) (32) (34) (36) (37) (43) (46)
Previous trauma	*	****	***		*	**	(33) (41) (42) (47) (48) (49) (50)
***Psychological and Behavioral Factors***							
Coping styles			*				(30)
Hyperactivity disorder		*	*		*		(46)
Sleep problems	**	***	***		*		(23) (28) (34) (35)
Resilience	*	***	***		****	*	(24) (30) (33) (36) (37) (42) (43) (44)
Low self-esteem	**	****	*			*	(22) (23) (27) (28) (41)
Long-term adaptation	*******	********	********		**		(21) (23) (25) (26) (27) (31) (33) (34) (35) (37) (39) (40) (47)
***Family and Social Support***							
Caregiver/family relationship	**	**	***	****		*	(29) (31) (32) (34) (36) (44) (50)
Lack of psychosocial support	***	********	******	*****		*	(20) (21) (22) (25) (26) (28) (32) (34) (44) (46) (49) (50) (51)
Receipt of psychological intervention	**	***	**	*			(20) (37) (48)
Volunteer aid	*	*					(20)
***Media and Information Exposure***							
Social media and news exposure	*	*		*			(20)
***Structural and Environmental Risk Factors***							
Violence		*	*		**	**	(24) (32) (33) (52)
Displacement	***	***	***		***	*	(22) (28) (29) (31) (34) (36) (43)
Substance use	*	**			**	**	(41) (43) (51)

#### Socio-Demographic & Personal Factors

Sociodemographic and personal factors associated with anxiety, depression, PTSD, stress, and suicidal ideation included female gender (Amos Nwankwo et al., 2021; Bianchini et al., 2017; Brown et al., 2021; Gerstner et al., 2020; Lai et al., 2020; Liang et al., 2023; Paul et al., 2015; Şam et al., 2025; Türkkan & Hatipoğlu, 2025; Wahab et al., 2021; Zhang et al., 2010), older adolescents compared with children under 15 years (Amos Nwankwo et al., 2021; Brown et al., 2021; Felix et al., 2011; Liang et al., 2023; Wahab et al., 2021) and low socioeconomic status (Dass-Brailsford et al., 2022; Gerstner et al., 2020; Lai et al., 2018; Liang et al., 2023; Wahab et al., 2021; Yakşi & Eroğlu, 2024; Zhang et al., 2010).

Only one of five studies that considered race/ethnicity reported an association between racial/ethnic minority status and higher levels of PTSD (Lai et al., 2018). Specifically, females and older adolescents were more likely to experience anxiety, depression, and PTSD. Low socioeconomic status — even when temporary due to post-disaster circumstances — was also associated with increased risk of PTSD and stress.

#### Exposure-Related Factors

Exposure-related factors differed by disaster type. Most of the literature focused on earthquakes, which were associated with high levels of anxiety, depression, PTSD, and suicidal ideation (Amos Nwankwo et al., 2021; Brown et al., 2021; Chen et al., 2021; Dass-Brailsford et al., 2022; Felix et al., 2011; Lai et al., 2020; Okuyama et al., 2017; Schwind et al., 2018; Vehid et al., 2006; Wahab et al., 2021; Zhang et al., 2010).

Exposure to hurricanes was linked to elevated levels of anxiety, depression, PTSD, and stress, compared with the baseline reported in each study (Chen et al., 2021; Lai et al., 2018; McLaughlin et al., 2010; Roberts et al., 2010; Şam et al., 2025; Yakşi & Eroğlu, 2024), while exposure to wildfires was associated with higher rates of suicidal thoughts and related mental health outcomes (Brown et al., 2019; Edwards et al., 2024; Gerstner et al., 2020; Lykins et al., 2023; McDonald-Harker et al., 2021).

However, flood exposure (Bianchini et al., 2017; E. D. Felix et al., 2020; McDonald-Harker et al., 2021) and tsunami exposure (Agampodi et al., 2011) were associated primarily with higher rates of depression and PTSD. In the case of flooding, one study also linked it to suicidal behaviors (McDonald-Harker et al., 2021).

Multiple exposures to natural disasters as well as prior trauma were linked to higher rates of depression and PTSD (Campbell et al., 2025; Chen et al., 2021; Danielson et al., 2017; Graham et al., 2017; Lykins et al., 2023; McDonald-Harker et al., 2021; Pazderka et al., 2021; Vehid et al., 2006; Yakşi & Eroğlu, 2024), as well as higher tendencies towards suicidal ideation.

Lastly, the loss of a relative/family member or property was associated with increased risk across all mental health outcomes, although only one study examined its association with suicide (McLaughlin et al., 2010).

#### Psychological and Behavioural Factors

Among the psychological and behavioral factors, resilience was identified as an important moderator of mental health outcomes, with lower resilience associated with higher levels of stress, depression, and PTSD, and to a lesser extent, anxiety and suicidal ideation (Agampodi et al., 2011; Amos Nwankwo et al., 2021; Chen et al., 2021; Dass-Brailsford et al., 2022; E. D. Felix et al., 2020; McDonald-Harker et al., 2021; McLaughlin et al., 2010; Vehid et al., 2006).

Sleep problems were described as a primary predictor of depression, although they were also indicative of anxiety, PTSD, and stress (Bianchini et al., 2017; Okuyama et al., 2017; Schwind et al., 2018; Wahab et al., 2021).

Long-term adaptation over time was associated with worse outcomes in terms of anxiety, depression, PTSD, and stress, according to the studies reviewed (Bianchini et al., 2017; Brown et al., 2019, 2021; Campbell et al., 2025; Chen et al., 2021; Edwards et al., 2024; Gerstner et al., 2020; Lai et al., 2018; Liang et al., 2023; Okuyama et al., 2017; Roberts et al., 2010; Şam et al., 2025; Schwind et al., 2018).

PTSD was reported to influence the development of coping styles (Dass-Brailsford et al., 2022) and was linked with hyperactivity disorder, with depression and stress also contributing as co-occurring or triggering factors (Graham et al., 2017).

Several studies linked anxiety, depression, and PTSD with low self-esteem in youth (Bianchini et al., 2017; Gerstner et al., 2020; Lai et al., 2020; Lykins et al., 2023; Wahab et al., 2021). However, only one of these studies linked low self-esteem to suicidal ideation.

#### Family and Social Support

Research has shown links between young people’s experiences of disasters and their relationships with family or caregivers, as well as the importance of social support. A lack of their family or caregiver support (Agampodi et al., 2011; Felix et al., 2011; Johnson et al., 2014; Lai et al., 2018; Schwind et al., 2018; Vehid et al., 2006; Yakşi & Eroğlu, 2024), as well as limited social support (Agampodi et al., 2011; Felix et al., 2011; Johnson et al., 2014; Lai et al., 2018; Schwind et al., 2018; Vehid et al., 2006; Yakşi & Eroğlu, 2024), was associated with increased level of anxiety, depression, PTSD, and stress compared with baseline or typical levels reported in each study.

Acting as a volunteer was linked to higher levels of anxiety and depression among young people (Zhang et al., 2010). On the other hand, receiving psychological interventions helped primarily reduce depression, as well as anxiety, PTSD, and stress (Danielson et al., 2017; Roberts et al., 2010; Zhang et al., 2010).

#### Media and Information Exposure

Although following news on traditional and social media was associated with greater life satisfaction, some mental health problems, such as anxiety, stress, and depression, were also intensified (Zhang et al., 2010).

#### Structural and Environmental Risk Factors

The displacement of affected residents was associated with a negative impact across all the mental health outcomes mentioned (Felix et al., 2011; E. D. Felix et al., 2020; Lai et al., 2018, 2020; Schwind et al., 2018; Vehid et al., 2006; Wahab et al., 2021). Similarly, substance use influenced the onset of all mental health problems except PTSD (E. D. Felix et al., 2020; Lykins et al., 2023; Prinstein et al., 1996).

Experiencing interpersonal violence was also considered a predisposing factor for suicidal ideation and stress, and to a lesser extent, was related to PTSD and depression (Amos Nwankwo et al., 2021; Chen et al., 2021; Yakşi & Eroğlu, 2024; Zuromski et al., 2019). Further details are provided in Table 4.

### Quality assessment

The NIH-QAT and CASP tools were used in accordance with the research design to evaluate the quality of the included studies, and the Mixed Methods Appraisal Tool (MMAT) was also employed due to the presence of mixed-methods studies. The studies were classified as cross-sectional (16), cohort (14), qualitative (1), and mixed-methods (2).

The overall quality scores of the evaluated papers were 10 out of 14 on the NIH-QAT checklist (**Supplementary Table 2**), 10 out of 10 on the CASP checklist (**Supplementary Table 3**), and 7 out of 7 on the MMAT (**Supplementary Table 4**). The NIH-QAT tool yielded evaluation scores ranging from 6 to 13, whereas the CASP and MMAT tools consistently received scores of 10 out of 10. Lastly, the assessed studies were categorized into three groups: good, fair, and poor (poor = ≤ 5; fair = 6–9; and good = ≥ 10). Overall, 21 studies were rated as “good” and 11 as “fair”.

## Discussion

### Mental Health Outcomes and Disaster Characteristics

Our findings indicate that the most frequently reported psychological impacts are anxiety, depression, and PTSD, although their intensity varies depending on the type of natural disaster described. Earthquakes were the most reported natural disasters.

In line with our findings, Campbell et al., 2025, highlight the distinctions between slow-onset catastrophes (e.g., drought) and sudden-onset disasters (e.g., hurricanes, earthquakes), reporting that sudden-onset events tend to cause greater emotional and social impacts to material and human losses. In contrast, slow-onset disasters may exert greater economic impact over time, which may be more psychologically manageable or allow better adaptation.

Similarly, Carnie et al., 2011 argued that drought negatively affects educational attainment and employment opportunities, contributing to stress and suicidal thoughts among youngpeople.

### Mental Health Outcomes and Contextual Variability

Our study further documented that mental health effects may persist in the long term, particularly among youth experiencing multiple exposures, family loss, or displacement.

Similar findings were reported by McLaughlin et al., 2010, who observed persistent symptoms of anxiety and PTSD in adolescents following Hurricane Katrina, and by Lai, B. S., & La Greca, A., 2020, who identified a higher prevalence of depression among youth displaced by floods in Asia.

However, some studies have shown divergent findings. For example, Johnson et al., 2014 found that adolescents exposed to disasters in the school context with prior prevention programs demonstrated high resilience and low psychological distress. Likewise, Prinstein et al., 1996 reported that adolescents indirectly exposed to Hurricane Hugo did not show a significant increase in PTSD symptoms compared to those not exposed.

These variations underscore the importance of considering contextual factors, preparedness levels, and exposure pathways when assessing the psychosocial impact of natural disasters on youth.

### Risk Factors for Youth Mental Health Outcomes

The results of this systematic review highlight multiple risk factors that amplify the negative impact of natural disasters on young people’s mental health. Across studies, female gender, older adolescence, low socioeconomic status, direct disaster exposure, displacement, and lack of psychosocial support were the most consistently associated with anxiety, depression, PTSD, stress, and suicidal behaviors.

Gender differences were identified as particularly notable. Female youth consistently reported higher levels of anxiety, depression, stress, suicidal thoughts, and PTSD, consistent with findings by Zhang et al., 2010, who attribute this difference to greater tendencies toward self-blame and internalizing coping styles.

However, this association was not uniform across all contexts. Vehid et al., 2006 observed an opposite pattern following an earthquake in Turkey, where males reported higher rates of suicidal ideation, possibly due to more externalizing coping styles.

These gendered coping patterns may also predispose males to substance use and behavioral risk. For example, Kanehara et al., 2016, reported increased alcohol use following the Great East Japan Earthquake and tsunami in north-eastern Japan, in March 2011, while Reijneveld et al., 2003 observed increased aggression and alcohol consumption among adolescents affected by a fire in Volendam, Netherlands, in January 2001.

Age has also been identified as a relevant factor, although findings remain mixed (Dass-Brailsford et al., 2022; Gerstner et al., 2020; Lai et al., 2018; Liang et al., 2023; Şam et al., 2025). Some previous studies claimed that adolescence, particularly early adolescence, is a critical developmental stage in which disaster exposure can have significant and lasting impacts on mental health (Gibbs et al., 2021; Grelotti et al., 2018; Levitt et al., 2012). Similarly, Kanehara et al., 2016, support the results of our study (Dass-Brailsford et al., 2022; Gerstner et al., 2020; Lai et al., 2018; Liang et al., 2023; Şam et al., 2025), which found greater vulnerability among adolescents than among children, as emotional symptoms tended to manifest more clearly later in life or even in adulthood.

Socioeconomic disadvantage emerged as another critical determinant. Disaster-related disruptions in access to food, water, and housing may worsen marginalization and resource disparities (Drolet et al., 2020; Grelotti et al., 2018), contributing to poorer mental health outcomes. This aligns with the finding of the present review (Bianchini et al., 2017; Brown et al., 2021; Gerstner et al., 2020; Gibbs et al., 2021; Lai et al., 2020; Masten & Narayan, 2012; Şam et al., 2025).

Such unequal access may also help explain why ethnic and racial minority groups may experience worsening mental health outcomes (Gibbs et al., 2021; Graham et al., 2017), as demonstrated by Lai et al., 2020. However, other studies that examined racial differences reported no significant disparities (Johnson et al., 2014; Pazderka et al., 2021; Reijneveld et al., 2003; Şam et al., 2025).

Worsening socioeconomic conditions were also linked to increased exposure to interpersonal violence and abuse – environments that heighten the risk of mental health disorders among youth (Amos Nwankwo et al., 2021; Chen et al., 2021; Reijneveld et al., 2003; Yakşi & Eroğlu, 2024). This relationship has been widely documented in the literature, with some authors suggesting that experiences such as sexual assault may pose a greater risk for PTSD than natural disasters themselves (McDermott & Cobham, 2014).

In addition to the speed of disaster onset, prior disaster experience (Campbell et al., 2025; Chen et al., 2021; Danielson et al., 2017; Johnson et al., 2014; Lykins et al., 2023; McDonald-Harker et al., 2021; Zuromski et al., 2019), as well as repeated exposure to multiple disasters (Campbell et al., 2025; Danielson et al., 2017; McDonald-Harker et al., 2021; Vehid et al., 2006; Yakşi & Eroğlu, 2024; Zuromski et al., 2019), were associated with increased adverse mental health outcomes, particularly depression and PTSD.

Furthermore, the lack of psychosocial support—whether familial, community, or institutional—was consistently identified as a cross-cutting risk factor. The loss of social networks and exposure to violence or abuse in post-disaster contexts also emerged as key predictors of mental health outcomes. Similar patterns have been reported McDermott & Cobham, 2014, who emphasize the importance of school-based interventions as safe spaces for emotional support.

### Resilience as a Dynamic and Contextual Process

Although resilience is often viewed as a protective factor, it was not always synonymous with mental well-being. In some contexts, resilience reflected forced or adaptive functioning under constrained conditions rather than sustained mental health.

In this systematic review, “acquired resilience” refers to resilience developed through lived experience, learning, and social support, rather than innate or genetic traits (Agampodi et al., 2011; Amos Nwankwo et al., 2021; Chen et al., 2021; Dass-Brailsford et al., 2022; E. D. Felix et al., 2020; McDonald-Harker et al., 2021; McLaughlin et al., 2010; Vehid et al., 2006).

In this regard, authors such as Theron et al., 2020, conclude that collaboration between youth and their social environments is essential for developing resilience, highlighting the need to conceptualize resilience as a contextual process rather than an individual trait. This is why other authors (Grelotti et al., 2018; Runkle et al., 2021) propose mental health programs and interventions that focus on building resilience.

Since early intervention programs have been shown to reduce levels of anxiety, depression, and PTSD (Danielson et al., 2017; Roberts et al., 2010; Zhang et al., 2010), efforts should focus on promoting stable environments, supportive relationships, and access to appropriate services. Personal resources, including resilience, encompass the ability to regulate emotions and behaviors, act proactively, and adopt a future-oriented perspective (Theron et al., 2020).

In line with this, Drolet et al., 2020, argue that resilience, rather than being an innate trait, is fostered through relational and social processes such as peer support and connection with caregivers or family members. This perspective supports the findings of our review (Agampodi et al., 2011; Brown et al., 2021; Felix et al., 2011; Graham et al., 2017; Johnson et al., 2014; Lai et al., 2018, 2020; Liang et al., 2023; Prinstein et al., 1996; Şam et al., 2025; Schwind et al., 2018; Vehid et al., 2006; Wahab et al., 2021; Yakşi & Eroğlu, 2024; Zhang et al., 2010; Zuromski et al., 2019).

However, only a small number of the included studies explicitly examined resilience in the context of youth mental health following natural disasters. Where addressed, resilience was primarily conceptualized at the individual level, often reflecting adaptive coping responses rather than sustained psychological well-being. This indicated a significant gap in the literature, particularly regarding collective, organizational, and structural forms of resilience that may have a crucial role in shaping youth mental health outcomes following disasters.

Therefore, future studies should include broader and more nuanced frameworks of resilience to better capture these multi-level dynamics.

### Implications for Policy and Practice

From a public health perspective, these findings underscore the urgent need to develop intersectoral policies that integrate psychosocial and mental healthcare services into disaster response systems, with a specific focus on young people. It is critical to implement accessible and culturally appropriate early intervention programs that include ongoing psychosocial support in both school and community settings.

Policies such as the integration of school-based mental health services, mobile counselling units for displaced youth, and coordinated referral systems that combine community services with disaster response teams should be considered. Furthermore, several authors (Klinner et al., 2023; Kranke et al., 2017; McDermott & Cobham, 2014) consider educational settings to be ideal spaces for delivering comprehensive, community-based approaches that are accessible and capable of reaching the majority of vulnerable and affected children and adolescents (Chen et al., 2021; Danielson et al., 2017; Graham et al., 2017; Prinstein et al., 1996; Roberts et al., 2010; Schwind et al., 2018; Wahab et al., 2021).

Accordingly, teacher and caregiver training, along with strengthening peer and family support networks, may help mitigate emotional impact and promote resilient environments. Similarly, McDermott & Cobham, 2014, advocate a tiered model that combines communication strategies and targeted clinical interventions with parent and teacher training, thereby overcoming the limitations of traditional models and optimizing the use of available resources.

Digital approaches such as crisis text messaging platforms (Runkle et al., 2021), which were implemented before and after Hurricane Florence, offer advantages in accessibility and 24-hour availability. These tools are likely to advance further with the support of artificial intelligence and may help reduce stigma - one of the key reasons many young people are reluctant to seek or accept help (Klinner et al., 2023; Kranke et al., 2017; Levitt et al., 2012).

Furthermore, strategies should prioritize populations with compounded vulnerabilities—such as displaced, low-income, or trauma-affected youth—to ensure equitable and sustainable responses. Investing in these measures may not only reduce immediate suffering but also prevent long-term chronic mental health disorders.

#### Limitations and Strengths

At the study level, since most included studies were cross-sectional, it was not possible to estimate the prior incidence of risk factors or establish causal relationships due to the lack of longitudinal follow-up. Furthermore, these studies may be subject to selection and recall biases, which can affect the accuracy of the findings, response rates, and the control of confounding factors.

In some cases, the data were derived from parent reported assessments of their children’s mental health rather than direct interviews with the youth. In other instances, the sample sizes were too small to ensure representativeness. Additionally, some studies did not sufficiently report comorbid conditions or specify baseline mental health status, which may limit the interpretation of the findings.

Although our review focused precisely on natural disasters, the distinction between natural and human-made hazards is increasingly blurred, as many events (e.g., cyclones, floods, heatwaves) are now intensified by human influences such as urbanization, climate change, and environmental degradation. This evolving overlap limits the traditional categorization used in our study and suggests that future studies may benefit from more nuanced or integrated disaster classifications.

At the review level, firstly, we were unable to perform a meta-analysis due to heterogeneity in reporting, measurement approaches, and study designs across the included studies. This limitation made quantitative synthesis unfeasible and may have introduced potential reporting bias.

Additionally, only English-language publications were included, and grey literature was excluded, which may have resulted in publication bias and the omission of relevant studies. Despite rigorous independent screening and data extraction by multiple reviewers, subjective judgment of reviewers during synthesis and categorization may have influenced the findings.

Lastly, the relatively small number of studies examining resilience and specific psychosocial interventions limits the generalizability of conclusions regarding protective factors and effective practices.

## Conclusions

In conclusion, natural disasters have significant and long-lasting consequences for the mental health of young people. The main impacts described in the reviewed studies included stress, anxiety, depression, PTSD, and suicidal ideation and attempts. To a lesser extent, some behavioral outcomes, such as hyperactivity and difficulties with concentration, were also reported.

This review also identified several consistent risk factors for poor mental health outcomes, including female gender, older adolescence, low socio-economic status, direct disaster exposure, displacement, bereavement, lack of social support, and substance use.

To address these vulnerabilities, a comprehensive, multisectoral approach involving schools, families, communities, and the healthcare system is required. Therefore, early mental health interventions, long-term monitoring, and inclusive community-based strategies are needed to reduce the psychological burden among youth in disaster-affected settings.

## Supplementary Information

Below is the link to the electronic supplementary material.

Supplementary Material (DOCX 5.18 MB)

## Data Availability

No datasets were generated or analysed during the current study.

## Declarations

### PROSPERO Registration Number

CRD42025648903.
