# Supplementary material for: Effect of Natural Disasters on Youth Mental Health: A Systematic Review
Source: J Child Adolesc Trauma. 2026 Mar 26;19(3):1665–79. doi: 10.1007/s40653-026-00853-y (PMC13546213; doi:10.1007/s40653-026-00853-y)
Supplement: Supplementary file 1 [file 40653_2026_853_MOESM1_ESM.docx]

**Supplementary Table 1: Key Terms Search Stratety**

| **Database** | **Search terms** | **Records** |
| --- | --- | --- |
| **PubMed** | (("natural disaster*") AND (mental health)) AND (youth*) - 74 Results 🡪 Filters: Free full text: 41 Results 🡪 Filtering by title reading: **20 COLLECTED** | **74** |
| **PsycINFO** | natural disaster AND TI mental health AND youth – 61 Results (many were duplicated by PubMeb) 🡪 Filtering by title reading: **28 COLLECTED** | **61** |
| **SCOPUS** | ( TITLE-ABS-KEY ( "natural disaster*" AND mental AND health ) AND TITLE-ABS-KEY ( youth* ) ) – 110 Results 🡪 Filters: Limited to All open access 🡪 47 RESULTS 🡪 Filtering by title reading: **12 COLLECTED** | **110** |
| **Web of Science** | ((TS=(natural disaster*)) AND TS=(mental health)) AND TS=(youth) – 257 Results 🡪 Refined By: All Open Access 🡪 87 RESULTS 🡪 Filtering by title reading: **37 COLLECTED** | **257** |

| **Supplementary Table 2** * Details on “other scales” used in the studies (n=33) | |
| --- | --- |
| **Type of scale for outcome measurement** | **References** |
| Patient Health Questionnaire, Adolescent version (**PHQ-A**) | (27) (41) |
| POST-TRAUMATIC GROWTH INVENTORY (**PTGI**) | (25) |
| Pittsburgh Sleep Quality Index | (35) |
| Posttraumatic Stress Disorder Self-Rating Scale (**PTSD-SS**) | (35) |
| Posttraumatic Stress Disorder Checklist (**PCL-5**) | (35) |
| Chinese version of the Screen for Child Anxiety Related Emotional Disorders (**SCARED**) | (34) (35) |
| Spence Children’s Anxiety Scale (**SCAS**) | (29) |
| Screen for Adult Anxiety Related Disorders (**SCAARED**) | (35) |
| Adolescent Self-Rating Life Events Checklist (**ASLEC**) | (35) |
| Life Satisfaction | (20) |
| Generalized Anxiety Disorder Screener (**GAD-7**) | (26) |
| Earthquake Exposure Assessment Tool for Children and Adolescents (**EEAT**) | (36) |
| PTSD module from National Survey of Adolescents (**NSA**) | (21) (50) |
| Beck Depression Inventory (**BDI**) Test | (38) |
| Depression module from National Survey of Adolescents (**NSA**) | (21) (50) |
| Social and Emotional Health Survey (**SEHS**) | (45) |
| Quick Inventory of Depressive Symptomatology (**QIDS-J**) | (37) |
| Flood Impact Questionnaire (**FIQ**) | (45) |
| Adjustment Disorder New Module 8 (**ADNM8**) | (43) |
| Impact of Fire Questionnaire (**IOF**) | (27) |
| **UNCOPE** Alcohol and Substance Abuse Screener | (43) |
| Brief Resilience Scale (**BRS**) | (43) |
| Brief symptom inventory | (28) |
| Environmental Threat Subscale of the Environmental Attitude Inventory | (43) |
| Adverse Childhood Experience Scale (**ACEs**) | (32) |
| KIDSCREEN QUESTIONNAIRE (**QUALITY OF LIFE**) | (27) |
| Life Events Checklist (**LEC**) | (32) |
| Short Mood and Feelings Questionnaire **(SMFQ)** | (32) |
| Center for Epidemiological Studies Depression Scale for Children (**CES-DC**) | (29) (32) |
| Psychological Distance Survey | (43) |
| Trauma Symptom Checklist for Children (**TSCC**) | (48) |
| Behavioral Indicator of Resiliency to Distress (**BIRD**) | (50) |
| Balloon Analog Risk Task-Youth (**BART-Y**) | (50) |
| Okasha Suicidality Scale | (29) |
| Tobacco Use Questionnaire | (41) |
| Rosenberg Self-Esteem Scale | (27) (41) |
| Kidscreen Questionnaire | (41) |
| Diagnostic Interview Schedule for Children (**DISC-IV**) | (31) |
| Achenbach Child Behaviour Checklist | (39) |
| Youth Self Report | (39) |
| Coping Scale (**CS**) | (22) |
| Mental Health Scale (**MHS**) | (22) |
| Children's Revised Impact of Event Scale-13 (**CRIES-13**) | (30) |
| Screen for Adolescent Violence Exposure (**SAVE**) | (33) |
| Hurricane Related Traumatic Experiences (**HURTE**) | (33) |
| Hurricane Harvey Impact questionnaire | (26) |
| Hurricane Exposure Questionnaire | (31) |
| Conflict Belief Questionnaire Short Form | (50) |
| Child Depression Inventory (**CDI**) | (23) (34) |
| Child Post-Traumatic Cognitions Inventory **(CPTCI)** | (30) |
| Parent Ratings Scales (**PRS**) | (33) |
| Impact of Events Scale-R (**IES**) | (28) (37) |
| Children’s posttraumatic response reaction index (**CPTS-RI**) | (34) |
| Behavioural Assessment System for Children, Second Edition (**BASC-2**) | (33) |
| Zung Self-Rating Anxiety Scale (**SAS**) | (25) (37) |
| Rural Adversity Mental Health Program (**RAMHP**) | (51) |
| Haiti Violence against Children Survey (**VACS**) | (24) |
| Avon Longitudinal Study of Parents and Children (**ALSPAC**) | (42) |

**Supplementary Table 3**

Summary of the National Institutes of Health Quality Assessment Tool (NIH-QAT) for Observational Cohort and Cross-Sectional Studies (n=32)

| **Studies** | **Assesment Criteria** | Research Questions | Study Population | Participation Rate | Inclusion criteria | Sample Size | Exposure prior to outcome | Sufficient timeframe | Different levels of exposure | Exposure measures | Multiple Exposure | Outcome Measures | Blinding of Outcomes | Loss of follow-up | Statistical analysis | Overall Quality |
| --- | --- | --- | --- | --- | --- | --- | --- | --- | --- | --- | --- | --- | --- | --- | --- | --- |
| Chen et al., 2021 | | YES | YES | YES | YES | YES | YES | YES | YES | YES | NA | YES | NA | NO | YES | GOOD |
| Türkkan T & Hatipoğlu E, 2024 | | YES | YES | YES | YES | YES | NO | NR | YES | YES | NO | YES | YES | NA | YES | GOOD |
| Schwind et al., 2018 | | YES | YES | NR | YES | NO | NO | NR | YES | YES | NA | YES | NR | NA | YES | FAIR |
| Paul et al., 2015 | | YES | YES | YES | YES | YES | NO | NR | YES | YES | NO | YES | NR | NA | YES | FAIR |
| Dass-Brailsford et al., 2022 | | YES | YES | YES | YES | NO | NO | NR | YES | NR | NR | YES | YES | NA | YES | FAIR |
| Brown et al., 2019 | | YES | YES | YES | YES | YES | NR | YES | YES | YES | NO | YES | YES | NA | YES | GOOD |
| Pazderka et al., 2021 | | YES | YES | YES | YES | YES | YES | YES | YES | YES | NA | YES | YES | NO | YES | GOOD |
| Agampodi et al., 2011 | | YES | YES | YES | YES | YES | NR | YES | YES | YES | YES | YES | YES | NA | YES | GOOD |
| Graham et al., 2017 | | YES | YES | NR | YES | YES | NR | YES | YES | YES | YES | YES | YES | NO | YES | GOOD |
| Roberts et al., 2010 | | YES | YES | YES | YES | NO | YES | YES | YES | YES | NO | YES | YES | NO | YES | GOOD |
| Zhang et al., 2010 | | YES | YES | YES | YES | YES | NO | NR | YES | YES | NO | YES | YES | NA | YES | GOOD |
| Liang et al., 2023 | | YES | YES | YES | YES | YES | NO | YES | YES | YES | NA | YES | YES | NO | YES | GOOD |
| Lai et al., 2020 | | YES | YES | NR | NR | YES | NO | YES | YES | YES | NA | YES | NR | NA | YES | FAIR |
| Campbell et al., 2025 | | YES | YES | YES | YES | YES | YES | YES | YES | YES | YES | YES | YES | NO | YES | GOOD |
| Lai et al., 2018 | | YES | YES | NR | NR | YES | NA | YES | YES | YES | NA | YES | YES | NR | YES | FAIR |
| Edwards et al., 2024 | | YES | YES | NA | NR | YES | YES | YES | YES | YES | YES | YES | YES | NA | YES | GOOD |
| Danielson et al., 2017 | | YES | YES | YES | YES | YES | NO | YES | YES | YES | NO | YES | YES | NO | YES | GOOD |
| Yakşi N & Eroğlu M, 2024 | | YES | YES | YES | YES | YES | NO | NR | YES | YES | NO | YES | YES | NR | YES | GOOD |
| Bianchini et al., 2017 | | YES | YES | NR | YES | YES | NO | YES | YES | YES | NO | YES | YES | NR | YES | GOOD |
| Amos Nwankwo et al., 2021 | | YES | YES | NA | NR | YES | NO | NR | YES | YES | NO | YES | YES | NR | YES | FAIR |
| Brown et al., 2021 | | YES | YES | YES | NR | YES | NO | YES | YES | YES | NO | YES | YES | YES | YES | GOOD |
| Lykins et al., 2023 | | YES | YES | NR | NR | YES | NO | YES | YES | YES | NO | YES | YES | NR | YES | FAIR |
| McDonald-Harker et al., 2021* | | YES | YES | NR | NR | YES | NO | YES | YES | NR | NO | YES | YES | NA | YES | FAIR |
| Şam et al., 2025* | | YES | YES | NR | YES | YES | NO | NR | YES | YES | NO | YES | YES | NR | YES | FAIR |
| Gerstner et al., 2020 | | YES | YES | NR | NR | YES | NO | YES | NR | NR | NO | YES | NR | NR | YES | FAIR |
| Felix et al., 2011 | | YES | YES | YES | YES | YES | NO | YES | YES | YES | NO | YES | YES | NR | YES | GOOD |
| Wahab et al., 2021 | | YES | YES | YES | YES | YES | NO | YES | YES | YES | YES | YES | YES | NR | YES | GOOD |
| Okuyama et al., 2017 | | YES | YES | YES | NR | YES | NO | YES | YES | YES | NO | YES | YES | YES | YES | GOOD |
| Zuromski et al., 2019 | | YES | YES | YES | YES | YES | NO | YES | YES | YES | YES | YES | YES | YES | YES | GOOD |
| Vehid et al., 2006 | | YES | YES | NR | NR | YES | NO | YES | YES | YES | NO | YES | YES | NR | YES | FAIR |
| Felix et al., 2020 | | YES | YES | NR | YES | YES | NO | YES | YES | YES | YES | YES | YES | NR | YES | GOOD |
| McLaughlin et al., 2010 | | YES | YES | YES | NR | YES | YES | YES | YES | YES | NA | YES | YES | YES | YES | GOOD |

Note: NA= Not Applicable; NR= Not Reported

**Supplementary Table 4**

Summary of Critical Appraisal Skills Programme (CASP) checklist (n=3)

| **Studies** | Was there a clear statement of the aims of the research? | Is a qualitative methodology appropriate? | Was the research design appropriate to address the aims of the research? | Was the recruitment strategy appropriate to the aims of the research? | Was the data collected in a way that addressed the research issue? | Has the relationship between researcher and participants been adequately considered? | Have ethical issues been taken into consideration? | Was the data analysis sufficiently rigorous? | Is there a clear statement of findings? | How valuable is the research? | Overall Quality |
| --- | --- | --- | --- | --- | --- | --- | --- | --- | --- | --- | --- |
| Carnie et al., 2011 | YES | YES | YES | YES | YES | YES | YES | YES | YES | YES | GOOD |
| McDonald-Harker et al., 2021* | YES | YES | YES | YES | YES | YES | YES | YES | YES | YES | GOOD |
| Şam et al., 2025* | YES | YES | YES | YES | YES | YES | YES | YES | YES | YES | GOOD |

**Supplementary Table 5** *Considering that the articles “Şam et al., 2025” and “McDonald-Harker et al., 2021”, are mixed method studies, in addition to having analyzed it using NIH-QAT and CASP tool, we will use the Mixed Methods Appraisal Tool (MMAT), version 2018

| **Studies** | **Category of Study Design** | **Methodological quality criteria** | **Responses** | | | |
| --- | --- | --- | --- | --- | --- | --- |
|  |  |  | Yes | No | Can’t Tell | Comments |
| Şam et al., 2025 | Screening questions  (for all types) | Are there clear research questions? | YES |  |  |  |
|  |  | Do the collected data allow to address the research questions? | YES |  |  |  |
|  |  | *Further appraisal may not be feasible or appropriate when the answer is ‘No’ or ‘Can’t tell’ to one or both screening questions.* | | | | |
|  | Mixed Methods | Is there an adequate rationale for using a mixed methods design to address the research question? | YES |  |  |  |
|  |  | Are the different components of the study effectively integrated to answer the research question? | YES |  |  |  |
|  |  | Are the outputs of the integration of qualitative and quantitative components adequately interpreted? | YES |  |  |  |
|  |  | Are divergences and inconsistencies between quantitative and qualitative results adequately addressed? | YES |  |  |  |
|  |  | Do the different components of the study adhere to the quality criteria of each tradition of the methods involved? | YES |  |  |  |
| McDonald-Harker et al., 2021 | Screening questions  (for all types) | Are there clear research questions? | YES |  |  |  |
|  |  | Do the collected data allow to address the research questions? | YES |  |  |  |
|  | Mixed Methods | Is there an adequate rationale for using a mixed methods design to address the research question? | YES |  |  |  |
|  |  | Are the different components of the study effectively integrated to answer the research question? | YES |  |  |  |
|  |  | Are the outputs of the integration of qualitative and quantitative components adequately interpreted? | YES |  |  |  |
|  |  | Are divergences and inconsistencies between quantitative and qualitative results adequately addressed? | YES |  |  |  |
|  |  | Do the different components of the study adhere to the quality criteria of each tradition of the methods involved? |  |  |  |  |
